# Evaluation of Anti-Inflammatory Effects of *Cannabis sativa* Extracts via Possible Modulation of mRNA Levels of Inflammatory Cytokines and Cannabinoid Receptors

**DOI:** 10.3390/nu18071106

**Published:** 2026-03-30

**Authors:** Joanna Bartkowiak-Wieczorek, Radosław Kujawski, Michał Szulc, Kamila Czora-Poczwardowska, Joanna Szymczak, Julia Gierszewska, Maria Miotk, Przemysław Mikołajczak, Edyta Mądry, Teresa Grzelak

**Affiliations:** 1 Physiology Department, Poznan University of Medical Sciences, 60-179 Poznan, Poland; jszymczak@ump.edu.pl (J.S.); emadry@ump.edu.pl (E.M.); tgrzelak@ump.edu.pl (T.G.); 2 Pharmacology Department, Poznan University of Medical Sciences, 60-806 Poznan, Poland; radkuj@ump.edu.pl (R.K.); mszulc@ump.edu.pl (M.S.); kczora@ump.edu.pl (K.C.-P.); przemmik@ump.edu.pl (P.M.); 3 Student Scientific Society of Physiology, Poznan University of Medical Sciences, 60-179 Poznan, Poland; 91227@student.ump.edu.pl; 4 Student Scientific Society of Pharmacology, Poznan University of Medical Sciences, 60-806 Poznan, Poland; marysiamiotk1@gmail.com

**Keywords:** non-steroidal anti-inflammatory drugs (NSAIDs), carrageenan, CB1 receptor, CB2 receptor, inflammatory cytokines, cyclooxygenases, COX-1, COX-2, cannabidiol (CBD)

## Abstract

**Background:** Low-THC *Cannabis sativa* L. extracts are commonly believed to offer potential alternatives to non-steroidal anti-inflammatory drugs (NSAIDs) for inflammatory pain management. This study evaluated the anti-inflammatory effects of two *C. sativa* extracts (Tygra, Dora) and pure cannabidiol (CBD) compared with acetylsalicylic acid (ASA) in a carrageenan-induced rat paw inflammation model. **Materials and Methods:** Fifty male Wistar rats were randomized into five groups: control, ASA (200 mg/kg), CBD (20 mg/kg), Extract B (Tygra), and Extract D (Dora). Treatments were administered intragastrically 30 min after carrageenan injection. Paw volume was measured at 0, 1, 3, 6, and 10 h, and mRNA levels of COX-1, COX-2, TNFα, NFκB, CB1, and CB2 were quantified by qPCR. **Results:** Unlike ASA, which reduced paw edema and NFκB expression at 10 h, CBD and both extracts increased edema compared to control. Specifically, Extract D induced greater edema than ASA, upregulated CB1 and CB2 (surpassing ASA CB2 levels), decreased TNFα, and reduced right-paw COX-2. Extract B increased edema (3 h vs. ASA), increased TNFα, and showed a positive COX-2/paw volume correlation. Furthermore, paw volume correlated negatively with CB2 under CBD treatment (which also lowered right-paw COX-2) and positively with COX-1 under ASA treatment. **Conclusions:** The results indicate that ASA has a clear anti-inflammatory effect, whereas CBD and the hemp extracts fail to inhibit—and may even exacerbate—the inflammatory response. Differences in endocannabinoid and inflammatory gene expression suggest extract composition–dependent modulation mechanisms.

## 1. Introduction

Inflammation is an evolutionarily conserved host defense response to tissue damage, pathogen infection, or exposure to irritants. It involves a complex cascade of molecular and cellular processes, including the activation of immune cells, the secretion of pro-inflammatory cytokines, and the synthesis of lipid mediators. However, uncontrolled or chronic inflammatory responses constitute a pathogenic mechanism that manifests as inflammatory pain [1].

Non-steroidal anti-inflammatory drugs (NSAIDs), including acetylsalicylic acid (ASA), remain the gold standard in the pharmacotherapy of inflammation. Their mechanism of action is primarily based on the inhibition of cyclooxygenase activity (COX-1 and COX-2), leading to reduced synthesis of prostaglandins responsible for the development of pain, edema, and fever. Despite high clinical efficacy, the long-term use of NSAIDs is associated with a significant risk of adverse effects, including gastrointestinal mucosal injury, nephrotoxicity, and an increased risk of cardiovascular complications [2].

This has spurred interest in alternative therapies and the search for new bioactive compounds, particularly those of plant origin derived from *Cannabis sativa*, known for their anti-inflammatory and analgesic properties and a potentially safer pharmacological profile [3].

In recent years, there has been growing interest in the endocannabinoid system (ECS) as an alternative target for modulating the inflammatory response. The ECS comprises cannabinoid receptors CB1 and CB2, endogenous lipid ligands, and enzymes responsible for their synthesis and degradation, and plays a key role in regulating the immune response, inflammatory processes, and oxidative stress. Particular importance is attributed to CB2 receptors, which are abundantly expressed on immune cells and whose activation inhibits pro-inflammatory cytokine secretion and restricts leukocyte migration to the site of inflammation [4].

Phytocannabinoids derived from *Cannabis sativa* L., particularly Delta (Δ) 9-tetrahydrocannabinol (THC) and cannabidiol (CBD), are significant modulators of the ECS. These compounds exhibit documented anti-inflammatory, immunomodulatory, and neuroprotective properties, mediated both through cannabinoid receptors and mechanisms independent of CB1/CB2 [5].

Importantly, CBD interacts with numerous signaling pathways associated with inflammation, including NFκB and PPARγ, and also modulates oxidative stress and microglial activity. These characteristics make the ECS a promising therapeutic target for the treatment of inflammatory diseases, especially in the search for safer alternatives to long-term NSAID use or at least as a pharmacological tool potentially enhancing the effects of this group of drugs [6].

Studies have demonstrated that CBD can reduce inflammation in carrageenan-induced paw edema in rats, a well-established model for investigation of acute inflammation, by modulating inflammatory cytokines such as COX-2 and TNFα and interacting with CB2 receptors [7]. However, the effects of low THC content whole-plant *Cannabis sativa* L. extracts on inflammatory pain and their molecular mechanisms remain underexplored [8].

This study aimed to evaluate the potential anti-inflammatory and analgesic effects of two low-THC *Cannabis sativa* L. extracts (Extract B and Extract D) in the above-mentioned rat model of carrageenan-induced inflammatory pain, comparing them with those of ASA (acetylsalicylic acid) and CBD. Additionally, the study investigated the modulation of mRNA levels of inflammatory mediators (COX-1, COX-2, TNFα), the signaling molecule NFκB and cannabinoid receptors (CB1, CB2) to elucidate the underlying mechanisms of action.

## 2. Materials and Methods

### 2.1. Chemicals

- Extract D (Hungarian cultivar KC Dora): 215.2 mg/g CBD and 13.3 mg/g Δ^9^-THC (Institute of Natural Fibres and Medicinal Plants, 60-630 Poznań, Polska).
- Extract B (Polish cultivar Tygra): 220.2 mg/g CBD and 15.5 mg/g Δ^9^-THC (Institute of Natural Fibres and Medicinal Plants).
- ASA (Acetylsalicylic acid, European Pharmacopoeia (EP) Reference Standard, Merck KGaA, Frankfurter Str. 250, 64293 Darmstadt, Germany, cat. no. A0200000).
- CBD (Cannabidiol solution, United States Pharmacopeia (USP) Reference Standard, Merc, Darmstadt, Germany, cat. no. 1089161).
- NaCl (Sodium chloride ACS reagent, ≥99.0%, Merc, cat. no. S9888).
- Carrageenan (Merc, cat. no. CAS 9000-07-1).
- Rapeseed oil (Pol-Aura Sp. z o.o., Zawroty, Poland, cat. no. R 8002-13-9).
- Transcriptor First Strand cDNA Synthesis Kit (Merck, Darmstadt, Germany, cat. no. 11483188001).
- Fast Start DNA Master SYBR Green I kit (Merck, cat. no. 4673484001).
- Primers for Real Time PCR (Novazym, 61-131 Poznan, Poland).

### 2.2. Preparation and Characterization of Cannabis sativa L. Extracts

Plant material was obtained from experimental cultivations (sowing and nitrogen fertilization at 30 kg/ha), and inflorescences were harvested at the late flowering stage [9]. The extraction procedure was performed in two stages: the plant material was treated with an organic solvent (n-hexane) at 30 °C for 30 min [9]. After centrifugation at 5500 rpm and appropriate dilution, the extract was vacuum concentrated at 50 mbar. The residue was subsequently dissolved in 96% (*v*/*v*) ethanol at 80 °C (ethanol:water system). Following re-evaporation of ethanol, the final decarboxylation step was performed at 130 °C for 30 min, converting the acidic precursor forms (CBDA and Δ^9^-THCA) to their pharmacologically active neutral counterparts (CBD and Δ^9^-THC) [9,10].

Two extract variants were used in the study, and their composition was analytically confirmed [9]. Residual concentrations of CBDA and Δ^9^-THCA were low in both variants, ranging from 0.15 to 1.2 mg/g.

Cannabinoid identification and quantification were performed using HPLC [10]. Samples (150 mg) were extracted in 10 mL of a methanol/THF mixture (30 min of shaking), followed by centrifugation at 5500 rpm. Measurements were conducted on a 10-fold diluted supernatant using a C18 column (gradient separation in 0.1% phosphoric acid solution and acetonitrile; UV detection at 230 nm) [11,12].

Chromatographic screening of 11 cannabinoids in *Cannabis sativa* extracts prepared from the same cultivar sources identified CBDV, CBDA, CBGA, CBG, CBD, CBN, Δ^9^-THC, CBNA, CBC, THCA, and CBCA. The most abundant component was CBDA (approximately 7%), followed by CBD (approximately 3%), with remaining cannabinoids present in trace amounts (0.04–0.39%) [10]. Terpene and flavonoid profiling were not performed in the current study and are planned for forthcoming investigations using GC-MS to elucidate the compositional basis of differential inflammatory responses between extracts.

### 2.3. Animals

The study utilized 50 male Wistar rats, aged 7–8 weeks, weighing 230–350 g. Rats were housed under controlled conditions (20 ± 2 °C, 55% humidity, 12 h light/12 h dark cycle) with unlimited access to water and laboratory feed. All procedures complied with local ethical regulations, No. 42/2015, and EU directives on laboratory animal welfare.

#### Sample Size Determination

The sample size was determined a priori using a statistical power analysis. Calculations were performed using the biomath.info/power online calculator [13] and independently verified using the statsmodels package in Python (v 0.14) with SciPy’s (v1.8) non-central F distribution.

The study employed a one-way analysis of variance (ANOVA) design with five independent experimental groups. For the primary analysis (one-way ANOVA, F-test), the following parameters were used: number of groups k = 5, significance level α = 0.05 (two-sided), target statistical power 1-β ≥ 0.80, numerator degrees of freedom df_1_ = 4, and denominator degrees of freedom df_2_ = 45. The power analysis was conducted for a range of Cohen’s f effect sizes (Table 1).

Results indicated that with *n* = 10 per group (total *n* = 50), the study achieves 55.4% power for a large effect (f = 0.40), 77.3% power for f = 0.50, and 91.5% power for f = 0.60. The minimum detectable effect size at 80% power was f = 0.515 (partial η^2^ = 0.210). For pairwise comparisons between any two groups (two-sample *t*-test, equal variances), the minimum detectable Cohen’s d at 80% power was 1.325, corresponding to a between-group mean difference of 1.33 standard deviations. With Bonferroni correction for 10 pairwise comparisons (α_adj_ = 0.005), the minimum detectable d increased to 1.835 (Table 2).

These effect sizes are consistent with the magnitudes of treatment effects commonly reported in comparable preclinical rat studies, where pharmacological, biochemical, or histopathological endpoints typically exhibit large between-group differences (f > 0.50; d > 1.0). Therefore, a sample size of *n* = 10 per group was deemed adequate to detect biologically and statistically meaningful differences while minimizing the total number of animals in accordance with the 3Rs principle and the ARRIVE 2.0 guidelines.

### 2.4. Inflammation Model

Experimental groups: Rats were randomly assigned to five groups (*n* = 10 per group). Before administration, the thickness of both paws was measured. The inflammation was induced in the rats using the carrageenan test similarly to one of our previous publications [12], which consisted of a single subcutaneous (s.c.) injection of 0.2 mL of a 1% carrageenan solution into the right hind paw 30 min before administration of the compounds. As a control, 0.2 mL of a 0.9% saline solution (NaCl) was injected once into the left hind paw in all animals. Thirty minutes after the injection of carrageenan and saline, the following were administered intragastrically (i.g.), depending on the remaining groups: rapeseed oil (vehicle), ASA, CBD, hemp spike extract variety B, and hemp inflorescence extract variety D.

### 2.5. Experimental Groups

- Group 1: Carrageenan + rapeseed oil (vehicle control).
- Group 2: Carrageenan + ASA (200 mg/kg).
- Group 3: Carrageenan + CBD (20 mg/kg).
- Group 4: Carrageenan + Extract B from *C. sativa* (20 mg/kg, variety Tygra).
- Group 5: Carrageenan + Extract D from *C. sativa* (20 mg/kg, variety Dora).

Treatments were administered, i.g, as a single dose, 0.5 h after carrageenan injection, with doses normalized to 1 mL per rat.

For groups no. 3–5, the dose of 20 mg/kg was calculated in relation to the cannabidiol content, as already mentioned in our previous publication [11]. This dosing strategy was adopted to enable direct comparison of the biological effects of pure CBD and full-spectrum extracts at equivalent CBD levels.

### 2.6. Measurement of Paw Swelling

Paw volume was measured using a plethysmometer (cat. no. 7140, Ugo Basile, 21036 Gemonio, Italy) at 0, 1, 3, 6, and 10 h post-treatment. Swelling was calculated as the difference in volume between the right (inflamed) and left (control) paws. The electrolyte solution (sodium chloride, NaCl) was prepared according to the manufacturer’s recommendations.

### 2.7. Biological Sampling

Post-experiment, rats were euthanized (decapitation by guillotine), and samples of blood and paw tissue were collected for biochemical and molecular analyses.

The paws collected (right and left from each individual) were immediately preserved in liquid nitrogen vapor for downstream procedures. The workstation was cleaned and sterilized immediately before the start of the biological material isolation procedure, and the entire area was cooled with liquid nitrogen vapor, on which the secured rat paws were placed. Each paw was then crushed with a sterilized hammer in a series of blows until it reached a powdery consistency, and the homogenate was immediately transferred (1 mL) into a commercial reagent for total RNA isolation, TriPure™ Isolation Reagent Kit (Roche Diagnostics GmbH, Sandhofer Strasse 116, 68305 Mannheim, Germany, catalog no. 11 667 165 001 (200 mL)). Further isolation procedures were then carried out, including extraction, centrifugation, drying, and dissolution in water, using a modified Chomczynski and Sacchi method [11,14].

### 2.8. Gene Expression Analysis

mRNA levels of cannabinoid receptors (CB1, CB2) and inflammatory molecules (COX-1, COX-2, TNFα) and NFκB were quantified using quantitative polymerase chain reaction (qPCR). Briefly, approximately 1–2 µg of total RNA was reverse transcribed using the Transcriptor First Strand cDNA Synthesis Kit according to the manufacturer’s protocol. The expression levels of COX-1, COX-2, and TNFα mRNA were analyzed using real-time PCR. The LightCycler TM system and the LightCycler Fast Start DNA Master SYBR Green I kit (LightCycler FastStart DNA Master SYBR Green I kit (Cat. No. 03 003 230 001/12239264001); Roche Diagnostics International Ltd., Rotkreuz, Switzerland) were employed for amplification. Quantification of mRNA levels was performed in 3 technical replicates. GAPDH served as the housekeeping gene (internal control) for normalization. The sequences of the primers used in the assay are listed as follows: COX-1: Primer-F: 5′ TCTATGCTGGTGGACTACG 3′, Primer-R: 5′ CATCTCCTTCTCTCCTGTG 3′; COX-2: Primer F: 5′ CTACGCCTGAGTTTCTGAC 3′, Primer R: 5′ ATTGTAAGTTGGTGGGCTG 3′; TNFα: Primer F: GCTCCCTCTCATCAGTTCC, Primer R: GCTTGGTGGTTTGCTACG; NFkB: Primer F: ACACCTCTACACATAGCAG, Primer R: CTACTCCCTCATCTTCTCC; GADPH: Primer-F: 5′ GATGGTGAAGGTCGGTGTG, 3 Primer-R: 5′ ATGAAGGGGTCGTTGATGG 3. The annealing temperature during the PCR amplification of individual amplicons ranged from 58 to 60 °C.

### 2.9. Statistical Analysis

Statistical analysis was performed using the Statistica 14 software (TIBCO Software Inc., Palo Alto, CA, USA) and PQStat 1.8.4 software (PQStat Software, Plewiska, Poland). The normality of the distribution of quantitative variables was checked with the Shapiro–Wilk test, and homogeneity of variance was tested using Levene’s test. For normally distributed data, one-way analysis of variance (ANOVA) followed by Tukey’s honestly significant difference (HSD) post hoc test and analysis of correlation (Pearson’s analysis) was applied. For non-normally distributed data, the Kruskal–Wallis test followed by the Bonferroni–Dunn post hoc test and analysis of correlation (Spearman’s analysis) was used.

Moreover, to compare differences in the gene expressions between right and left rat paws, the dependent samples *t*-test and Wilcoxon matched-pairs test (adequate for parametric and non-parametric data distributions) were applied, and to compare changes in variables in the analyzed rat groups during a few hours after the carrageenan test and rapeseed oil, ASA, CBD, Extract B or Extract D, with the Friedman test, followed by the Bonferroni–Dunn post hoc test. The statistically significant level of error was established at α < 0.05. Bonferroni corrections were applied in the cases of multiple comparisons (to control type I errors). Continuous variables are presented in the figures as medians with quartile ranges (Q1 and Q3).

## 3. Results

### 3.1. Analysis of Changes in Differences Between Rat Paws (Right and Left)—The Volumes (V) of Rat Paws (Right and Left)

**Figure 1:**
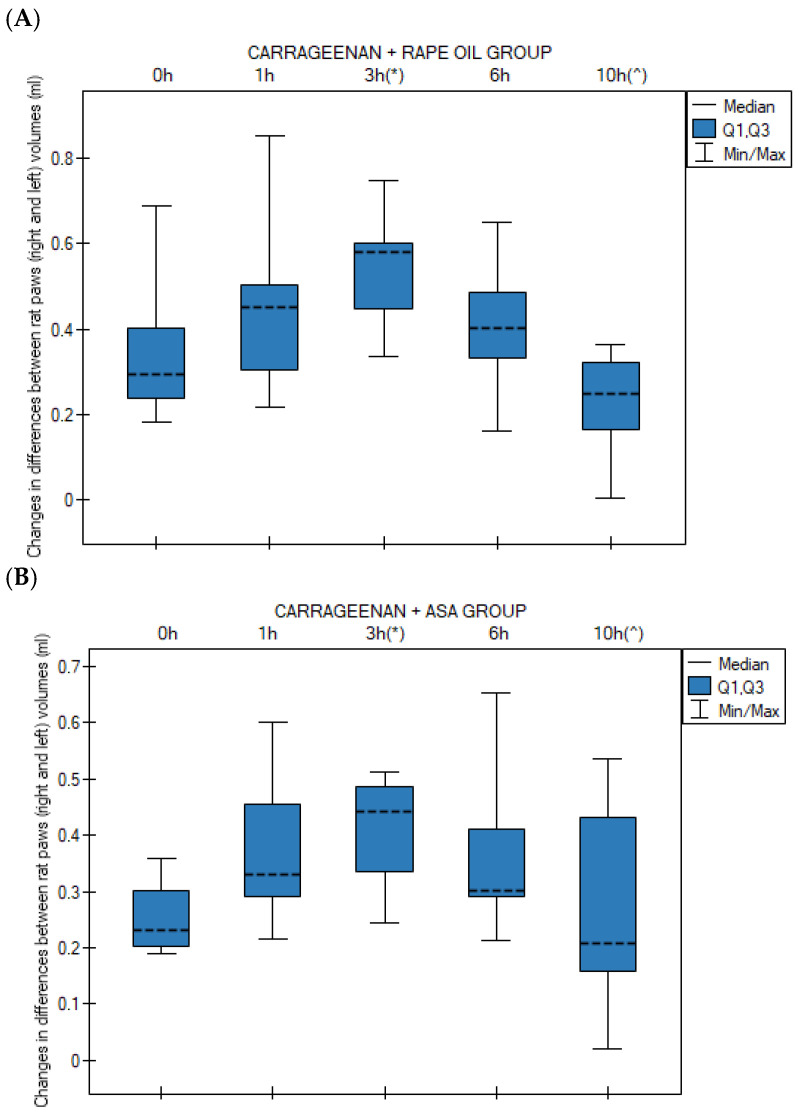

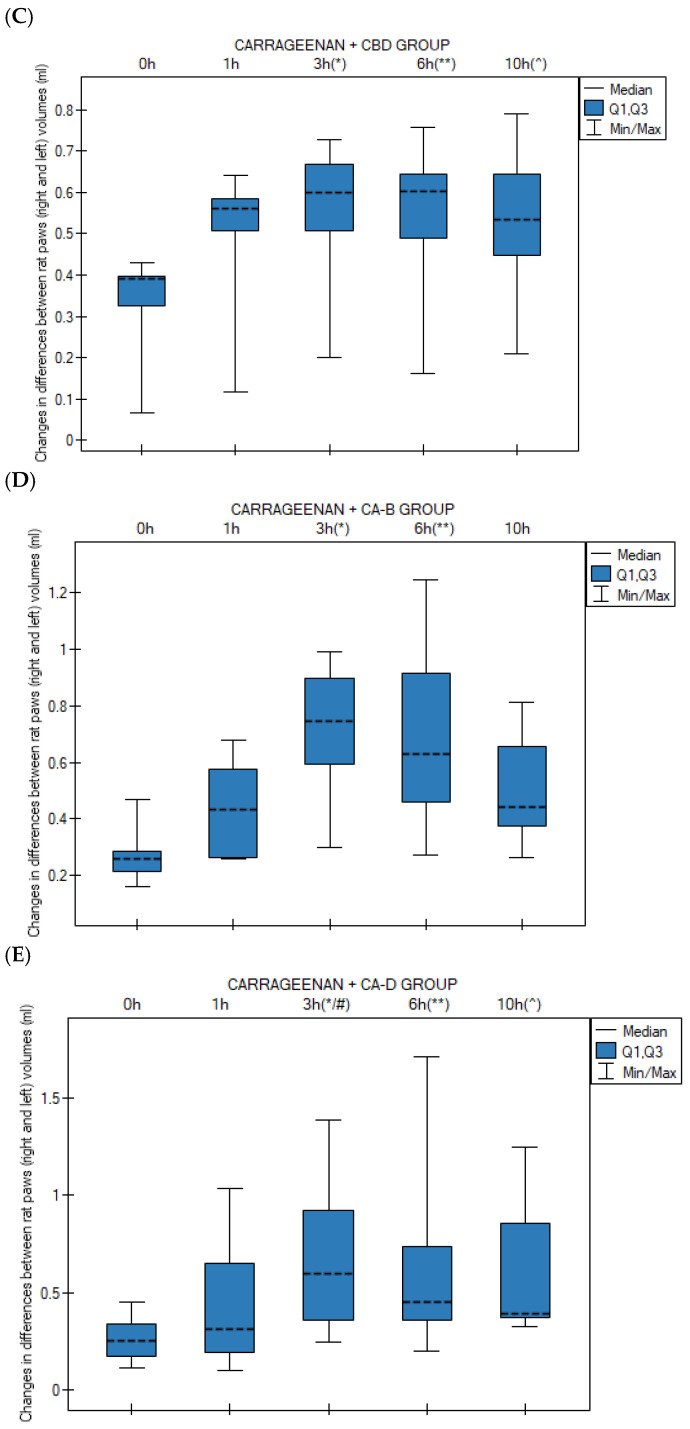
Changes in differences between rat paws (right and left) volumes (V) during the experimental time at 0 h (V0h), 1 h (V1h), 3 h (V3h), 6 h (V6h) and 10 h (V10h) in the Friedman test followed by the Bonferroni–Dunn post hoc test after: (**A**) rapeseed oil (i.g.) in rat group (*n* = 10) with carrageenan test; (*) *p* = 0.047 for V3h vs. V0h; (^) *p* = 0.001 for V10h vs. V3h; (**B**) ASA (acetylsalicylic acid) (i.g.) in rat group (*n* = 10) with carrageenan test; (*) *p* = 0.024 for V3h vs. V0h; (^) *p* = 0.019 for V10h vs. V3h; (**C**) CBD (cannabidiol) (i.g.) in rat group (*n* = 10) with carrageenan test; (*) *p* = 0.0008 for V3h vs. V0h; (**) *p* = 0.002 for V6h vs. V0h; (^) *p* = 0.024 for V10h vs. V0h; (**D**) CA-B—Extract B—*Cannabis sativa* L. extract, variety Tygra (i.g.) in rat group (*n* = 10) with carrageenan test; (*) *p* = 0.00002 for V3h vs. V0h; (**) *p* = 0.0004 for V6h vs. V0h; (**E**) CA-D—Extract D—*Cannabis sativa* L. extract, variety Dora (i.g.) in rat group (*n* = 10) with carrageenan test; (*) *p* = 0.0004 for V3h vs. V0h; (**) *p* = 0.002 for V6h vs. V0h; (#) *p* = 0.019 for V3h vs. V1h; (^) *p* = 0.011 for V10h vs. V0h; n—number of rats; i.g.—intragastric administration; data presented as medians (---) with quartile ranges (Q1, Q3).

In the control group, a statistically significant increase in edema occurred at the 3rd hour compared to time 0 h (*p* = 0.047). At the 10th hour, a significant decrease in edema was observed compared to the peak at the 3rd hour (*p* = 0.001) (Figure 1A). In the Carrageenan + ASA group, the profile of changes was similar to that of the control group. A significant increase in edema relative to baseline (0 h) occurred at the 3rd hour (*p* = 0.024). Similarly to the control group, a significant decrease in edema volume occurred at the 10th hour relative to the 3rd hour (*p* = 0.019) (Figure 1B).

In contrast to the ASA and control groups, in the group receiving CBD, edema persisted at a significantly higher level compared to time 0 h throughout most of the experiment: at the 3rd hour (*p* = 0.0008), 6th hour (*p* = 0.002), and still at the 10th hour (*p* = 0.024). No statistically significant decrease was noted at 10 h relative to the peak of inflammation (Figure 1C). In the Carrageenan + Extract B group, a significant increase in inter-paw volume difference (ΔG) relative to time 0 h was observed at the 3rd hour (*p* = 0.00002) and at the 6th hour (*p* = 0.0004) (Figure 1D).

The Carrageenan + Extract D group demonstrated a pronounced progression of edema. A significant increase occurred as early as between the 1st and 3rd hour (*p* = 0.019). Relative to time 0 h, significantly higher ΔG (the change in paw thickness or volume, serving as a quantifiable measure of the developing edema) values were observed at the 3rd hour (*p* = 0.0004), 6th hour (*p* = 0.002), and 10th hour (*p* = 0.011). As with CBD, there was no evidence of inflammation resolution at the 10th hour (Figure 1E).

### 3.2. Group-by-Group Comparison of Inter-Paw Differences in Hind Paw Volume Changes (Right vs. Left) Across Time Points

No statistically significant differences in changes in paw volume (between the right and left hind paw) were observed among rats at the 0 h, 1 h, and 6 h time points across all studied groups: (1) rapeseed oil, (2) ASA, (3) CBD, (4) Extract B and (5) Extract D in the applied test.

**Figure 2:**
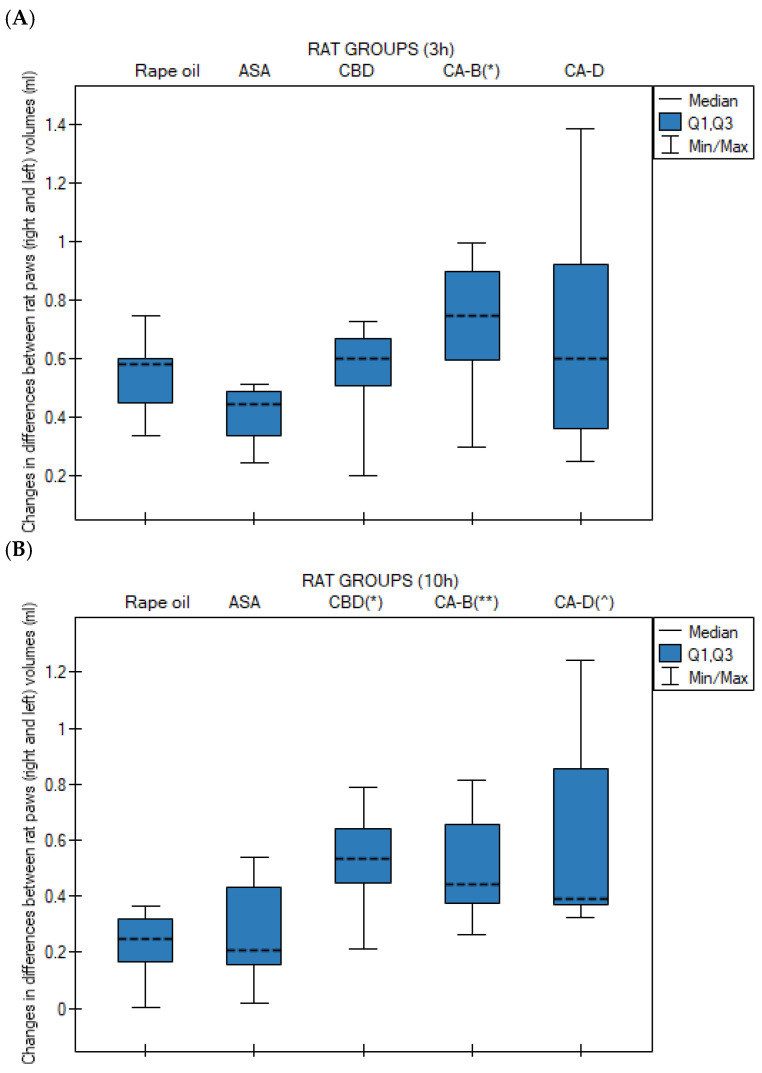
Changes in differences between rat paws (right and left) volumes in Kruskal–Wallis test followed by Bonferroni–Dunn post hoc test at 3 h (3 h) (**A**) and 10 h (10 h) (**B**), after rapeseed oil (*n* = 10), ASA (acetylsalicylic acid, *n* = 10), CBD (cannabidiol, *n* = 10), Extract B—*Cannabis* sativa L. extract, variety Tygra (*n* = 10) and Extract D—*Cannabis sativa* L. extract, variety Dora (*n* = 10) (i.g.): (**A**) (*) *p* = 0.013 for Extract B vs. ASA, (**B**) (*) *p* = 0.009 for CBD vs. rapeseed oil; (**) *p* = 0.028 for Extract B vs. rapeseed oil; (^) *p* = 0.023 for Extract D vs. rapeseed oil. *n*—number of rats; i.g.—intragastric administration; data presented as medians (---) with quartile ranges (Q1, Q3).

At 3 h (the peak of the inflammatory response), a statistically significant difference was observed (*p* = 0.013) between group 4 (Extract B) and group 2 (ASA). Extract B showed a higher median edema volume than the ASA group (Figure 2A).

At 10 h, all cannabinoid-treated groups exhibited significantly higher edema levels compared to the control group (rapeseed oil): group 3 (CBD) vs. group 1 (oil), *p* = 0.009; group 4 (Extract B) vs. group 1 (rapeseed oil), *p* = 0.028; and group 5 (Extract D) vs. group 1 (rapeseed oil), *p* = 0.023 (Figure 2B).

### 3.3. Changes in Right and Left Paw Volume at Different Time Points

**Figure 3:**
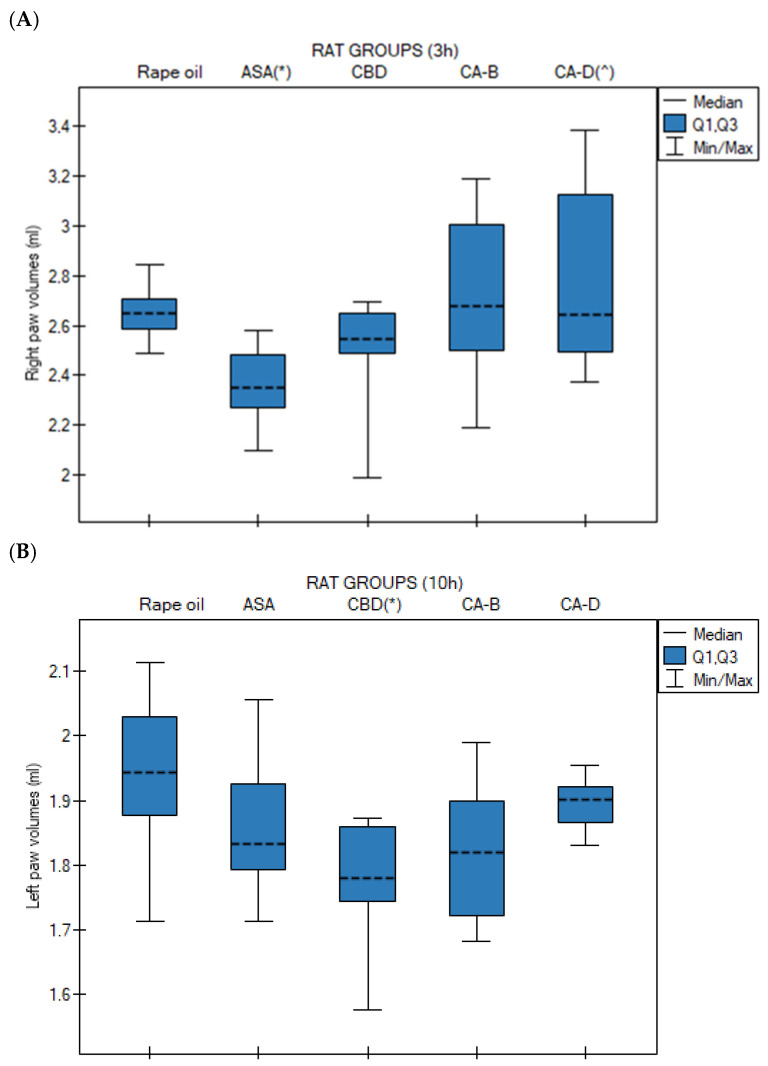
Medians (with Q1, Q3 quartiles) of rat right paws volumes after rapeseed oil (*n* = 10), ASA (acetylsalicylic acid, *n* = 10), CBD (cannabidiol, *n* = 10), Extract B—*Cannabis sativa* L. extract, variety Tygra (*n* = 10) and Extract D—*Cannabis sativa* L. extract, variety Dora (*n* = 10) (i.g.) in rat with carrageenan test; in Kruskal–Wallis test followed by Bonferroni–Dunn post hoc test (**A**) Right paw at 3 h (3 h)—(*) *p* = 0.014 for ASA vs. rapeseed oil; (^) *p* = 0.027 for Extract D vs. ASA; (**B**) Left paw at 10 h (10 h)—(*) *p* = 0.020 for CBD vs. rapeseed oil. n—number of rats; i.g.—intragastric administration.

Medians (with Q1 and Q3 quartiles) of right (Figure 3A) and left paw (Figure 3B) volumes in rats at 0 h, 1 h, 3 h, 6 h, and 10 h after administration of rapeseed oil, CBD, Extract B, and Extract D in the carrageenan-induced paw edema test were analyzed using the Kruskal–Wallis test followed by Bonferroni–Dunn post hoc test.

No statistically significant differences were observed at 0 h, 1 h, and 6 h for the left paw and at 0 h, 1 h, 3 h, and 6 h for the right paw.

For the right paw, the ASA group exhibited the lowest median paw volume, compared to the control group (*p* = 0.014). Extract D induced a significantly higher median right paw volume compared to the ASA group (*p* = 0.027) (Figure 3A). In contrast, for the left paw, the CBD group showed a significantly lower median paw volume at 10 h compared to the control group (rapeseed oil, *p* = 0.020) (Figure 3B).

### 3.4. Expression Levels of Cannabinoid Receptors and Inflammatory Factors

#### 3.4.1. Expression Levels of CB1, CB2, COX-1, COX-2, TNFα, and NFκB Genes in Right Paws Versus Left Paws in Analyzed Groups

Paired analysis of carrageenan-challenged animals revealed no significant differences in the transcription levels of CB1, CB2, COX-1, COX-2, TNFα, and NFκB genes between the right and left hind paws in the control ASA and Extract B groups (*p* > 0.05).

**Figure 4:**
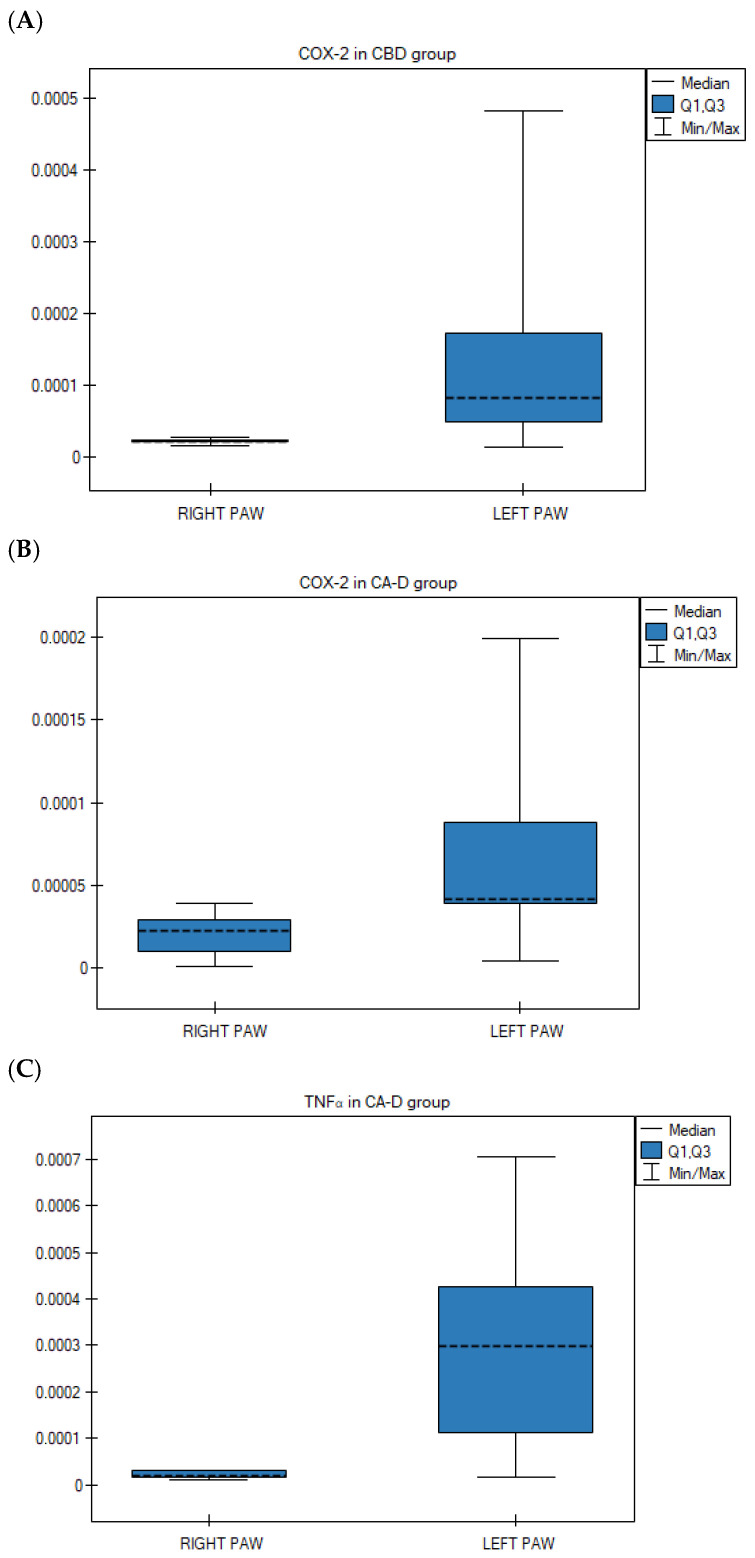
Gene expression in right paws and left paws in the rat group with carrageenan test: (**A**) For COX-2 after CBD (cannabidiol) (i.g.), *p* = 0.028 (non-parametric data—in Wilcoxon matched-pairs test); (**B**) For COX-2 after Extract D—*Cannabis sativa* L. extract, variety Dora (i.g.) *p* = 0.021 (non-parametric data—in Wilcoxon matched-pairs test) (**C**) For TNFα after Extract D—*Cannabis sativa* L. extract, variety Dora (i.g.) *p* = 0.013 (parametric data—ANOVA test for paired data) i.g. intragastric administration; data presented as medians (---) with quartile ranges (Q1, Q3).

However, statistically significant differences were observed in two other treatment groups. Specifically, COX-2 gene expression was significantly lower in the right paws compared to the left in the CBD group (*p* = 0.028; Wilcoxon matched-pairs signed-rank test) (Figure 4A) and the Extract D group (*p* = 0.021; Wilcoxon matched-pairs signed-rank test) (Figure 4B). Furthermore, TNFα gene expression was significantly lower in the right hind paws compared to the left following the administration of Extract D (*p* = 0.013; ANOVA for paired data) (Figure 4C).

#### 3.4.2. Expression Levels of CB1, CB2, COX-1, COX-2, TNFα, NFκB Genes in Right Paws in Analyzed Groups

**Figure 5:**
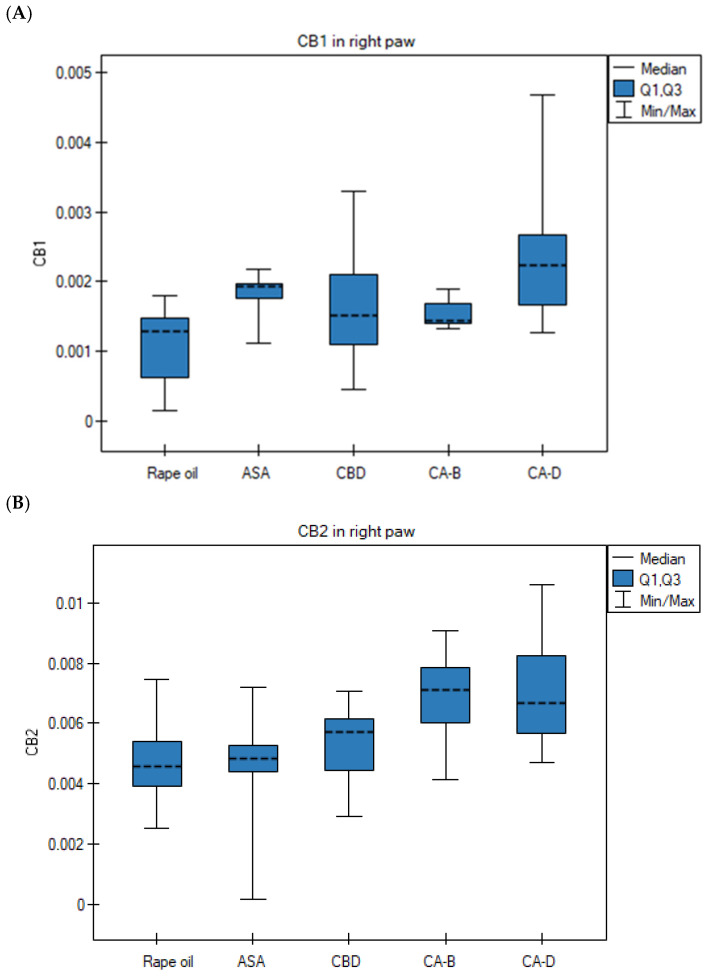

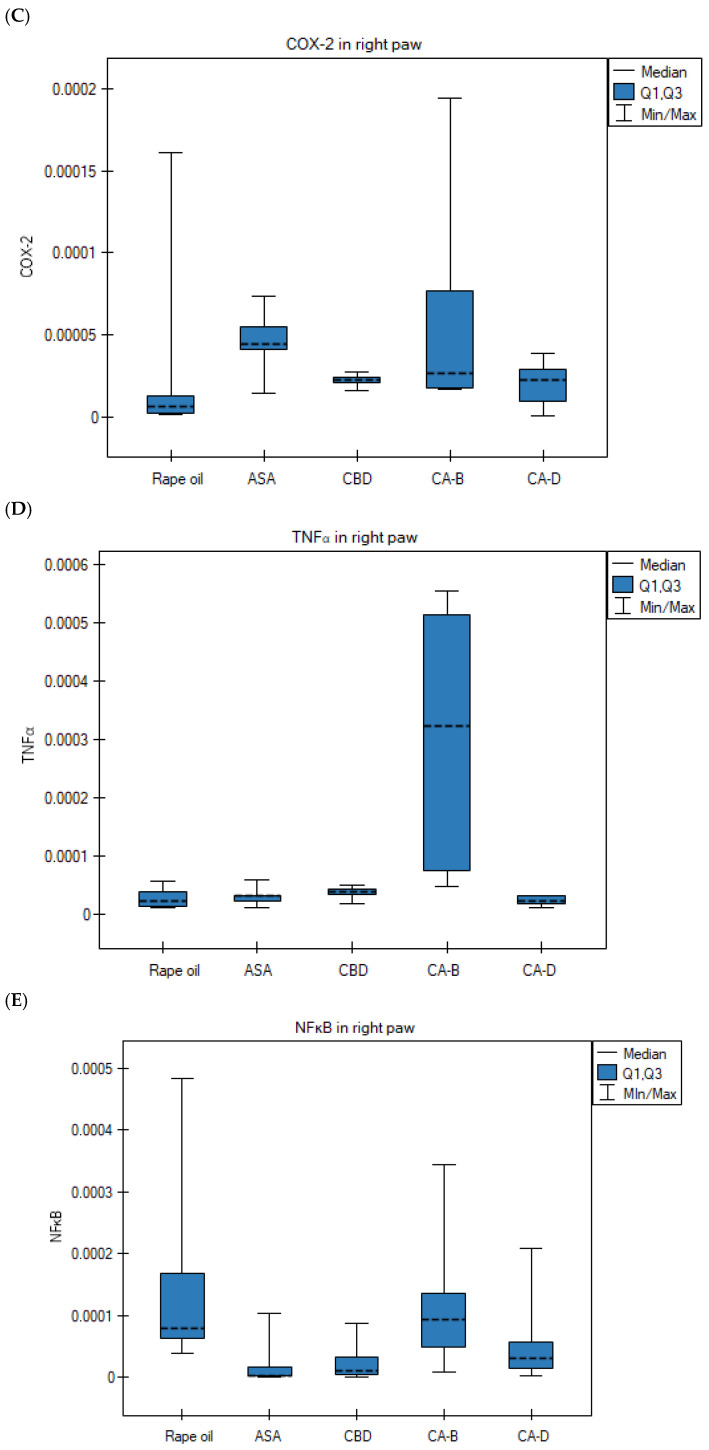
Gene expressions in right paws after rapeseed oil (i.g.) in the rat group with carrageenan test, after ASA (acetylsalicylic acid) (i.g.) in the rat group with carageenan test, after CBD (cannabidiol) (i.g.) in the rat group with carrageenan test, after Extract B—*Cannabis sativa* L. extract, variety Tygra (i.g.) in the rat group with carrageenan test, after Extract D—*Cannabis sativa* L. extract, variety Dora (i.g.) in the rat group with carrageenan test; (**A**) for CB1, *p* = 0.0022 in Extract D vs. rapeseed oil group (in ANOVA test, followed by Tukey post HOC test); (**B**) for CB2, *p* = 0.045 in the right paw for Extract D vs. rapeseed oil group (in ANOVA test, followed by Tukey post hoc test, parametric data); *p* = 0.023 in the right paw for ASA vs. Extract D group (in ANOVA test, followed by Tukey post hoc test; parametric data) (**C**) for COX-2, *p* = 0.0022 in the right paw for ASA group vs. rapeseed oil group in the Kruskal–Wallis test, followed by the Bonferroni–Dunn post hoc test, non-parametric data); (**D**) for TNFα, *p* = 0.0001 in the right paw for Extract B group vs. rapeseed oil group in ANOVA test, followed by Tukey post hoc test parametric data; *p* = 0.0001 in the right paw for Extract B vs. ASA group in ANOVA test, followed by Tukey post hoc test, parametric data; *p* = 0.0001 in the right paw for Extract B vs. CBD group in ANOVA test, followed by Tukey post hoc test parametric data; *p* = 0.0001 in the right paw for extract B group vs. Extract D group in ANOVA test, followed by Tukey post hoc test, parametric data; (**E**) for NFκB, *p* = 0.0052 in the right paw for the ASA group vs. the rapeseed oil group in the Kruskal–Wallis test, followed by the Bonferroni–Dunn post hoc test, non-parametric data; i.g.—intragastric administration; data presented as medians (---) with quartile ranges (Q1, Q3).

The level of CB1 mRNA in the Extract D group (right paw) was significantly higher than in the rapeseed oil group (ANOVA, Tukey post hoc test, *p* = 0.0022) (Figure 5A). The CB2 gene expression in the right paws in the Extract D group was significantly higher than in the rapeseed oil group (*p* = 0.045) and the ASA group (*p* = 0.023) (ANOVA, Tukey post hoc test) (Figure 5B). Its transcript level in the right paws in the ASA group was significantly higher than in the rapeseed oil group (Kruskal–Wallis test, Bonferroni–Dunn post hoc test, *p* = 0.0022) (Figure 5C). The level of TNFα mRNA in the right paws in the Extract B group was significantly higher than in the rapeseed oil group (*p* = 0.0001), the ASA group (*p* = 0.0001), the CBD group (*p* = 0.0001), and the Extract D group (*p* = 0.0001) (ANOVA, Tukey post hoc test) (Figure 5D). The NFκB mRNA level in the right paws in the ASA group was significantly lower than in the rapeseed oil group (Kruskal–Wallis test, Bonferroni–Dunn post hoc test, *p* = 0.0052) (Figure 5E).

The transcription profile of the CB1, CB2, COX-1, and NFκB genes in the left paws was similar across the five experimental groups, with no statistically significant differences observed (*p* > 0.05).

**Figure 6:**
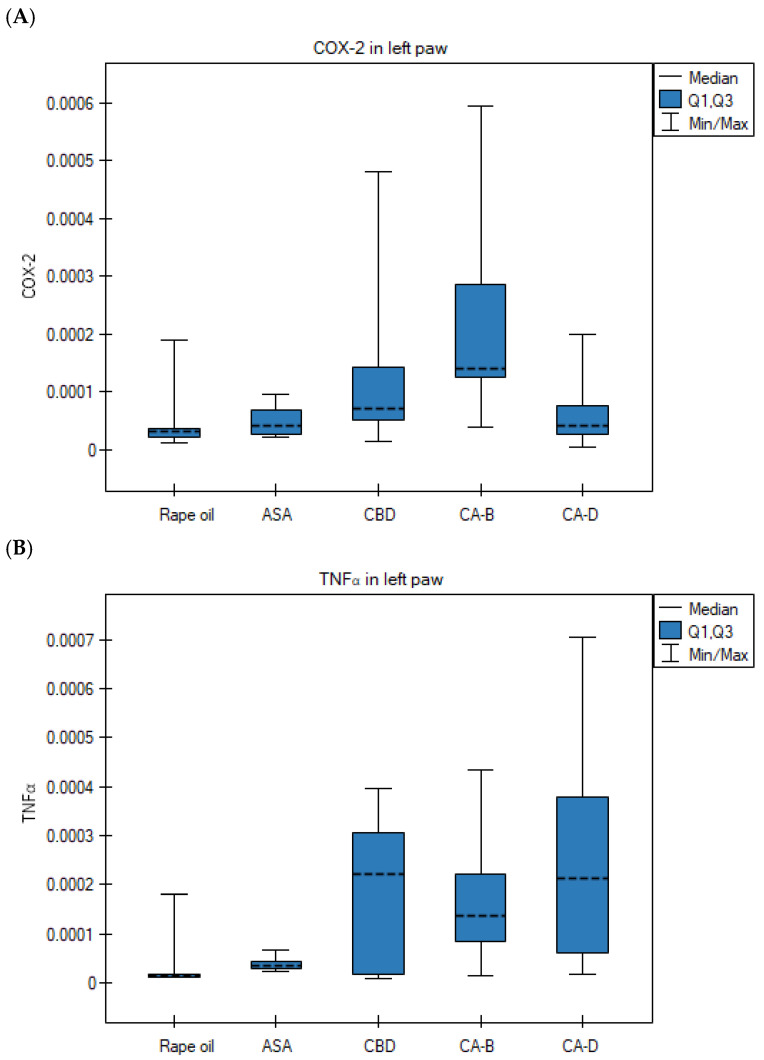
Gene expression in left paws after rapeseed oil (i.g.) in the rat group with carrageenan test, after ASA (acetylsalicylic acid) (i.g.) in the rat group with carrageenan test, after CBD (cannabidiol) (i.g.) in the rat group with carrageenan test, after Extract B—*Cannabis sativa* L. extract, variety Tygra (i.g.) in the rat group with carrageenan test, after Extract D—*Cannabis sativa* L. extract, variety Dora (i.g.); (**A**) for COX-2, *p* = 0.010 in the right paw for the Extract B vs. the rapeseed oil group in the Kruskal–Wallis test, followed by the Bonferroni–Dunn post hoc test; non-parametric data (**B**) for TNFα, *p* = 0.0057 for Extract D group vs. rapeseed oil group in the Kruskal–Wallis test, followed by the Bonferroni–Dunn post hoc test; non-parametric data i.g.—intragastric administration; data presented as medians (---) with quartile ranges (Q1, Q3).

However, the COX-2 mRNA level in the left paws in the Extract B group was significantly higher than in the rapeseed oil group (Kruskal–Wallis test, Bonferroni–Dunn post hoc test, *p* = 0.010) (Figure 6A). The TNFα gene expression in the left paws in the Extract D group was significantly higher than in the control group (Kruskal–Wallis test, Bonferroni–Dunn post hoc test, *p* = 0.0057) (Figure 6B).

### 3.5. Spearman Correlation Analysis (Non-Parametric Distributions)

Spearman correlation analysis revealed, in the CBD group, negative correlations between right paw volume at 3 h and CB2 expression, as well as between right paw volume at time point 0 and NFκB expression.

In the CA-B group correlations were observed between COX-2 transcription in the right paw and paw volume at 3 h (V3h), changes in the volume difference between both paws (ΔG) at 3 h and 6 h (V6h), and right paw volume at 10 h (V10h). In addition, a positive correlation was demonstrated between NFκB mRNA level in the right paw and the change in the volume difference between paws (ΔG) at time point 0 (V0h) (R_S_ = 0.9, *p* = 0.037) (Table 3).

Positive correlations were observed in the right paw of rats in the control group between COX-2 and NFκB transcripts levels, in the ASA group between CB2 and NFκB mRNAs, in the CA-B group between COX-1 and COX-2, and in the CA-D group between TNFα and NFκB (Table 4).

### 3.6. Pearson Correlation Analysis (Parametric Distributions)

Pearson correlation results in the right paw showed positive correlations in the control group between CB1 and CB2 mRNA levels, in the ASA group between CB2 and COX-1 mRNAs, and in the CBD group between CB1 and COX-1 transcripts (Table 5).

Positive Pearson correlations were noted between right paw volume and TNFα mRNA level in the oil-treated group at 3 h; between changes in the volume of both paws at 6 h and TNFα transcripts in the control group; in the ASA group between changes in the volume of both paws at 3 h and COX-1 mRNA level; in the ASA group between changes in the volume of both paws at 1 h and COX-2 mRNAs; as well as between right paw volume in the ASA group at 6 h and COX-2 transcription profile, and between right paw volume in the ASA group at 10 h and TNFα mRNAs (Table 6).

Negative Pearson correlations were observed in the right paw between paw volume and CB1 mRNA level at 0 h in the CBD group, between right paw volume and COX-1 transcription profile at 0 h in the CBD group, between right paw volume and COX-1 mRNAs at 1 h in the Extract B group, and between changes in volume of the right versus left paw at 3 h and COX-1 transcripts in the CA-B group (Table 7).

## 4. Discussion

The rat paw edema model induced by carrageenan injection is one of the most well-validated models of acute inflammation [15]. In our experiment, we observed the classical biphasic response in the control group (rapeseed oil). The first phase (0–2.5 h), mediated by the release of biogenic amines such as histamine, serotonin, and bradykinin [16], was characterized by progressive edema of the right paw following carrageenan administration in all groups. The second phase (3–6 h), characterized by COX-2-induced prostaglandin overproduction and the release of pro-inflammatory cytokines such as TNFα and IL-1β [16], occurred at 3 h (increase in paw volume, *p* = 0.047 vs. 0 h), consistent with literature describing this model in Wistar rats [17].

The difference in volume between the inflamed and control paws served as a key parameter for assessing the inflammatory response, reducing inter-individual variability and enabling direct evaluation of the effects of the tested substances. In our study, no significant differences were observed between groups at 0 h and 1 h, indicating comparable baseline conditions and the absence of a substance effect during the early phase of inflammation. A key observation in the control group was a statistically significant decrease in edema at 10 h (*p* = 0.001 vs. 3 h), suggesting a spontaneous resolution phase. The statistically significant increase in edema at 3 h relative to baseline (*p* = 0.024) and its decrease at 10 h compared to the peak (*p* = 0.019) confirm that the carrageenan model relied on prostaglandin synthesis during the studied time window [18].

Pearson correlation analysis in the control group showed a positive correlation between paw volume at 3 h and TNFα mRNA expression, in line with reports identifying TNFα as a key cytokine driving edema and neutrophil recruitment in this model [19]. Additionally, a positive Spearman correlation was observed between COX-2 and NFκB, known for directly inducing PTGS2 (prostaglandin-endoperoxide synthase 2, a synonym to COX2) gene transcription in response to inflammatory stimuli [20].

At the molecular level, inflammation was further confirmed by increased mRNA transcription of CB1 and CB2 receptor genes in the inflamed right paw of animals receiving Extract D, compared to controls, reflecting a protective mechanism aimed at limiting hyperalgesia and edema [21]. CB2 activation in macrophages and neutrophils typically inhibits the release of pro-inflammatory mediators [22]. Extract D more effectively stimulated ECS receptor overexpression than external CBD, suggesting an entourage effect in which other phytocannabinoids and, possibly, terpenes synergistically enhance the endocannabinoid response [23]. In the CBD treated group, a negative Spearman correlation was observed between paw volume at 3 h and CB2 mRNA level, supporting the protective role of CB2 component in peripheral tissues, especially when considering the fact that the CB2 agonism inhibits leukocyte infiltration and cytokine release, directly reducing inflammatory exudate [22].

A similar inflammatory pattern to the control group was observed in the ASA group. Edema increased until 3 h and then significantly decreased at 10 h, confirming the validity of the model. ASA results align with classical irreversible COX inhibitor pharmacodynamics, reflecting its ability to reduce prostaglandin synthesis and attenuate edema in the carrageenan model [24]. Positive Pearson correlations between paw volume and COX-2 mRNA expression at 6 h were observed, along with a strong positive Spearman correlation between CB2 and NFκB transcript levels. The high COX-2 transcription in ASA-treated animals may appear paradoxical but can be explained by compensatory upregulation of COX-2 mRNA following enzymatic inhibition [25]. The positive CB2–NFκB correlation suggests that increased levels of CB2 transcripts (expression) may buffer NFκB activity, representing a protective mechanism despite ASA treatment [22,26]. Paradoxically, ASA also induced a significant increase in COX-2 mRNA compared to controls, consistent with feedback-driven compensatory transcription [27]. Nevertheless, ASA effectively reduced NFκB mRNA levels, confirming its anti-inflammatory genomic effect.

While the literature emphasizes the anti-inflammatory effects of CBD and other phytocannabinoids, including reductions in TNF, IL1, and IL6 [28], and oral CBD has been shown to reduce carrageenan edema in both phases [17], our results revealed more complex dynamics. In the CBD group, a negative Spearman correlation was found between paw volume at 0 h and NFκB transcripts, suggesting early pathway suppression consistent with the results presented by Nicholas and Kaplan [29]. A positive Pearson correlation between CB1 and COX-1 mRNA levels in this group may indicate nociceptive interactions, in which CB1 modulates inflammatory pain via constitutive prostanoids [30]. Significantly lower COX-2 mRNA expression in the inflamed paw compared to the contralateral paw in the CBD and Extract D groups supports local anti-inflammatory action via suppression of COX-2 transcription below physiological levels, consistent with cannabinoid-mediated MAPK/p38 inhibition [7]. An interesting observation in our study was the significantly lower median paw volume in the left (control) paw in the CBD group at the 10 h time point compared with the control group. Although saline injection does not induce the acute inflammatory cascade characteristic of carrageenan, the injection procedure itself and the introduction of fluid into the plantar space elicit local tissue microtrauma and transient mechanical edema. The accelerated return to baseline volume observed in the CBD-treated group suggests a potent systemic anti-edematous effect of this compound. This finding aligns with the existing literature, which demonstrates that systemically acting CBD effectively expedites the resolution of inflammation and facilitates tissue fluid resorption, even following mild mechanical injuries [7,17].

Extract D reduced TNFα mRNA level in the inflamed paw relative to the healthy paw, highlighting that plant chemotype (THC: CBD ratio, terpene profile) can determine whether a preparation acts suppressively or immunostimulatory on cytokine pathways [31]. In contrast, Extract B showed pro-inflammatory activity, increasing TNFα mRNA in the right paw compared to all other groups. This may reflect pro-inflammatory or irritant components, or a terpene profile that enhances the early immune response. The pro-inflammatory effects were evident from sharp edema increases at 3 h, maintained at 6 h, and persistently elevated edema at 10 h following Extract D. Spearman correlations in the Extract B group revealed links between COX-2 mRNA profile and paw volume at 3 h and changes in both paws, suggesting a possible shift toward mediators characteristic of the resolution phase (e.g., 15d-PGJ2 prostaglandins) [32]. Negative Pearson correlations between paw volume changes and COX-1 transcripts further underscore the complexity of extract-mediated modulation, distinct from separately administered CBD, consistent with an entourage effect [33]. At 3 h, the Extract B group exhibited significantly greater edema than ASA-treated animals, indicating a lack of anti-inflammatory activity during the acute phase.

Paradoxically, at 10 h, all cannabinoid-receiving groups showed greater paw volume differences than controls, suggesting that these substances did not reduce edema and may have contributed to its maintenance or exacerbation in the late phase. Discrepancies with the literature may reflect differences in experimental conditions, extract formulation, administration route, or interactions with other extract components. It is possible that terpenes or fatty acids in the extracts enhance pro-inflammatory responses, outweighing CBD’s anti-inflammatory effects [34]. These findings raise the hypothesis that extract components may modulate the resolution phase of inflammation or partially sustain it, warranting further compositional and mechanistic analyses. Although preclinical literature generally attributes immunomodulatory or anti-inflammatory properties to cannabinoids and terpenes [35], other evidence suggests that terpenes may exert only transient suppression or complex modulation of inflammatory responses [36].

The paradoxical increase in paw edema observed across three groups (CBD-administered group, Extract B-administered group and Extract D-administered group) at 10 h post-carrageenan warrants further explanation of the mechanism of action. One potential explanation lies in the well-documented bell-shaped dose–response curve characteristic of CBD pharmacology. Gallily et al. [36,37] demonstrated that CBD exhibits maximal anti-inflammatory efficacy at intermediate doses (approximately 5 mg/kg intraperitoneally), with diminished effects at both lower and higher doses [37]. Given the low oral bioavailability of CBD (approximately 6% in the fasted state) [38], the effective systemic CBD concentration achieved after intragastric administration of 20 mg/kg may have placed the response on the subtherapeutic limb of this inverted U-shaped curve [38]. Alternatively, it cannot be ruled out that the achieved concentration was sufficient to activate pro-inflammatory pathways (e.g., via TRPV1/TRPV2 agonism) without reaching the threshold required for anti-inflammatory CB2-mediated effects. Qin et al. demonstrated that CBD acts as a potent TRPV2 agonist (EC50 = 3.7 µM), triggering dose-dependent CGRP release from dorsal root ganglion neurons [39], while McKenna and McDougall showed that cannabinoid-mediated activation of TRPV channels can promote neurogenic inflammation via substance P and CGRP release [40].

Furthermore, the divergent inflammatory profiles of Extract B and Extract D underscore the importance of the entourage effect hypothesis in cannabinoid pharmacology. While the concept posits that whole-plant preparations may exert enhanced therapeutic effects through synergistic interactions among cannabinoids, terpenes, and flavonoids [33], recent systematic reviews have revealed that evidence for a consistent, predictable entourage effect remains limited and context-dependent [41,42]. In fact, the term “contra-entourage effect” [42] has been proposed to describe situations in which extract components interact antagonistically, potentially diminishing or reversing the beneficial effects of individual cannabinoids [42,43,44]. Our finding that full-spectrum extracts did not outperform pure CBD, and in fact tended to sustain edema, is consistent with this emerging paradigm. Blevins et al. demonstrated that individual *Cannabis*-derived terpenes exert differential immunomodulatory effects on human primary leukocytes, with α-pinene showing anti-inflammatory properties while limonene had no effect [41]. Downer et al. further noted that certain *Cannabis* terpenes may produce transient immunostimulatory rather than immunosuppressive effects, depending on concentration and cellular context [42].

The question of causality in the observed correlations between paw volume and gene expression represents a fundamental interpretive challenge. The positive Pearson correlation between paw volume at 3 h and TNFα mRNA in the control group may reflect either TNFα-driven edema formation through vascular permeability enhancement and neutrophil recruitment (primary driver), or tissue-injury-induced transcriptional upregulation secondary to tissue swelling and cellular stress (secondary response) [45]. Both mechanisms likely operate simultaneously in a positive feedback loop. Similarly, the negative correlation between paw volume and CB2 expression in the CBD group at 3 h may reflect either a protective CB2 response limiting edema severity or, alternatively, suppression of CB2 transcription by advanced inflammation. Resolving this directionality requires time-resolved gene expression studies (sampling at 1, 3, 6, and 10 h), pharmacological intervention with selective antagonists, and protein-level validation approaches planned for forthcoming studies.

The authors plan to conduct a long-term observational study extending the monitoring period to 24–72 h post-carrageenan to determine whether cannabinoid preparations delay the resolution phase or actively sustain inflammation. Additionally, the interaction between pro-resolving lipid mediators (lipoxins, resolvins) and the endocannabinoid system, as demonstrated by Pamplona et al., who showed that lipoxin A4 acts as an allosteric enhancer of CB1 [46], warrants investigation in the context of cannabinoid-mediated modulation of inflammation resolution.

### Strengths and Limitations of the Study

This study was based on a well-validated rat paw carrageenan edema model, characterized by a biphasic inflammatory response. The model’s reliability was confirmed by results from the control group, which showed the expected spontaneous resolution of inflammation at 10 h. Model validity was further supported by the response in the reference group treated with acetylsalicylic acid (ASA), where classical edema reduction and inhibition of the NFκB pathway were observed, consistent with the dynamics reported in the literature.

A key strength of this study is its comprehensive methodological approach, combining standard phenotypic assessment (plethysmometry) with molecular analysis of gene expression (qPCR). Measurement of transcriptional profile of genes encoding key inflammatory mediators (COX-1, COX-2, TNFα, NFκB) and cannabinoid receptors (CB1, CB2) allowed us to capture dissociations between the lack of a visible clinical effect (edema reduction) and significant modulation of intracellular signaling pathways.

The study’s value is further enhanced by the comparative analysis of pure CBD and two *Cannabis sativa* plant extracts (Tygra and Dora varieties) with distinct phytochemical profiles. This approach provided insights into the potential entourage effect and the possible combined action of phytochemicals in full-spectrum preparations. Additionally, the application of correlation analyses (Pearson and Spearman) enabled the detection of non-obvious mechanistic relationships, such as links between cannabinoid receptor expression (measured by assessing changes in the transcript profile for CB1 and CB2) and the severity of edema.

Certain methodological limitations of this study should be acknowledged, as they may have influenced the observed results. The compounds were administered intragastrically (i.g.). Cannabinoids given via this route exhibit low and variable bioavailability and are subject to a strong first-pass effect. The lack of measurement of active compound concentrations in the blood prevents a definitive assessment of whether the absence of a therapeutic effect was due to the mechanism of action or insufficient tissue levels.

Only a single dose of cannabinoids (20 mg/kg) was used in this study. Given the often-observed nonlinear (bell-shaped) dose–response relationship in cannabinoid pharmacology, the lack of a broader dose range is a significant limitation. It cannot be excluded that the chosen dose was subtherapeutic or, conversely, too high, potentially resulting in a paradoxical pro-inflammatory effect.

A major challenge in interpretation is the divergent results observed, suggesting that cannabinoids in this model may have interfered with the natural resolution of inflammation or acted irritant-like. This is particularly relevant to the ambiguous profile of Extract B, which induced a systemic and significant increase in pro-inflammatory TNFα, exceeding levels observed in all other groups.

The study’s results are based on mRNA analysis (qPCR). The lack of verification at the protein level (e.g., via ELISA or Western blot) constitutes a limitation, as changes in gene expression do not always translate linearly to functional protein levels.

Future investigations should incorporate protein-level assessments (Western blot, ELISA, or immunohistochemistry) and functional enzymatic assays (e.g., COX-2 activity via prostaglandin E2 quantification) to bridge the gap between transcriptional and functional outcomes.

Furthermore, given the conservative nature of the Bonferroni correction applied to multiple comparisons across five experimental groups (used to limit the risk of type I error) and the relatively modest sample size (*n* = 10 per group), there is an inherent risk of type II error, particularly for subtle gene expression differences [45]. We are also aware that non-significant results should therefore be interpreted with caution and do not necessarily indicate the absence of biologically meaningful effects. Future studies with larger sample sizes (n ≥ 15–20 per group) and complementary statistical approaches (e.g., false discovery rate control via Benjamini–Hochberg procedure) are warranted to capture potentially meaningful but statistically underpowered effects observed in the present dataset.

For future studies we are also considering the use of selective receptor antagonists, i.e., TRPV1 antagonists (e.g., capsazepine), CB1 antagonists (e.g., rimonabant/AM251), and CB2 antagonists (e.g., AM630) to validate the proposed receptor-mediated mechanisms. Detailed chromatographic profiling of terpene and minor cannabinoid content of both extracts is planned, and dose–response assessments (e.g., 5, 10, 20, 40, and 80 mg/kg CBD) should be included to fully characterize the anti-inflammatory dose-effect relationship of these preparations.

Taking into account the strengths and limitations of this study, these findings highlight the complexity of cannabinoid pharmacology in the context of inflammatory processes and provide a clear direction for future investigations to unravel their immunomodulatory mechanisms, optimize delivery strategies, and translate molecular effects into clinically relevant outcomes in the management of inflammation.

## 5. Conclusions

This comparative study revealed fundamental differences in the pharmacodynamic profiles of intragastrically administered *Cannabis* preparations (extracts from *Cannabis sativa* L. varieties Tygra and Dora) and CBD compared to acetylsalicylic acid (ASA). We confirmed inhibition of the cyclooxygenase pathway, manifested as a significant reduction in edema in the ASA group during the peak phase of the inflammatory response (3 h). During this same window, dominated by prostaglandin release and COX-2 activity [15], none of the studied *Cannabis* preparations demonstrated therapeutic efficacy. Moreover, in the Extract B group, edema levels were significantly higher than in the ASA group, which, upon oral administration, may indicate insufficient bioavailability of active compounds during the acute phase or a lack of direct effect on eicosanoid synthesis.

Particular attention should be paid to the phytocannabinoid activity profile in the late phase of the experiment (10 h). Contrary to the expected resolution of inflammation, all *Cannabis*- treated groups (CBD, Extract B and Extract D) showed statistically significant persistence, and even enhancement, of paw volume differences compared to the control group. This finding contradicts the prevailing paradigm of exclusively anti-inflammatory cannabinoid action and prompts the formulation of a new research hypothesis. We suggest that the effect observed at 10 h resulted from a biphasic mechanism of cannabinoid action on the vascular and nervous systems. Given that CBD and other phytocannabinoids act as agonists of vanilloid receptors (TRPV1), prolonged activation may lead to the release of pro-inflammatory neuropeptides, such as substance P and calcitonin gene-related peptide (CGRP), promoting a neurogenic component of edema during a phase in which classical inflammatory mediators should already be degraded [7,17].

Alternatively, the observed results may indicate interference of exogenous cannabinoids with physiological resolution pathways of inflammation. This process is active, regulated by lipoxins and resolvins, and its disruption can promote the transition of acute inflammation to a chronic state. The lack of a pronounced “entourage effect” in our study—where full-spectrum extracts showed no advantage over the CBD and even tended to sustain edema—suggests that the complex plant matrix may contain compounds that further burden metabolic pathways involved in mediator clearance upon oral administration.

In summary, oral administration of *Cannabis sativa* L. extracts and CBD did not exhibit anti-edematous effects in the acute carrageenan model, and in later phases may delay the return to tissue homeostasis. Considering these results, future studies should focus on elucidating the molecular basis of this phenomenon, particularly by assessing levels of pro-inflammatory mediators and applying selective TRPV1 and CB1 receptor antagonists, also taking into account other known components of *C. sativa* L. extracts. These results also caution against extrapolating the anti-inflammatory properties of cannabinoids to acute inflammatory conditions following systemic oral administration.

## Figures and Tables

**Table 1:** ANOVA power analysis (k = 5, *n* = 10/group, α = 0.05).

Cohen’s f	Effect Category	Power (1-β)	Adequate
0.10	Small	0.074 (7.4%)	No
0.25	Medium	0.231 (23.1%)	No
0.40	Large	0.554 (55.4%)	No
0.50	-	0.773 (77.3%)	No
0.515	Min. detectable	0.800 (80.0%)	Yes
0.60	—	0.915 (91.5%)	Yes
0.80	—	0.996 (99.6%)	Yes

**Table 2:** Pairwise *t*-test power (*n* = 10/group, α = 0.05).

Cohen’s d	Effect Category	Power (1-β)	Adequate
0.20	Small	0.071 (7.1%)	No
0.50	Medium	0.185 (18.5%)	No
0.80	Large	0.395 (39.5%)	No
1.00	-	0.562 (56.2%)	No
1.33	Min. detectable	0.800 (80.0%)	Yes
1.50	-	0.887 (88.7%)	Yes
2.00	-	0.988 (98.8%)	Yes

**Table 3:** Spearman correlations (in the case of the right paw) for the mRNA level of genes versus volumes/changes in volume (in the case of the right paw).

Group	Variable 1	Variable 2	Rs	*p*-Value
CBD	Paw volume at 3 h (right paw)	CB2 expression (right paw)	−0.738	0.037
CBD	Paw volume at 0 h (right paw)	NFκB expression (right paw)	−0.829	0.042
Extract B (Tygra)	Paw volume at 3 h (right paw)	COX-2 expression (right paw)	−0.900	0.037
Extract B (Tygra)	Δ Paw volume (right–left) at 3 h	COX-2 expression (right paw)	−0.900	0.037
Extract B (Tygra)	Paw volume at 6 h (right paw)	COX-2 expression (right paw)	−0.900	0.037
Extract B (Tygra)	Δ Paw volume (right–left) at 10 h	NFκB expression (right paw)	0.900	0.037

CBD—cannabidiol, Extract B (CA-B)—*Cannabis sativa* L. extract, variety Tygra, Rs—Spearman correlation coefficient (for non-parametric data distributions), *p*—statistical significance level, Δ paw volume—difference between right and left paw volumes.

**Table 4:** Spearman correlations for the expression of genes.

Group	Variable 1	Variable 2	Rs	*p*-Value
Control (rapeseed oil)	COX-2 expression (right paw)	NFκB expression (right paw)	0.733	0.016
ASA	NFκB expression (right paw)	CB2 expression (right paw)	0.833	0.010
Extract B (Tygra)	COX-1 expression (right paw)	COX-2 expression (right paw)	0.900	0.037
Extract D (Dora)	TNFα expression (right paw)	NFκB expression (right paw)	0.786	0.021

Extract B (CA-B)—*Cannabis sativa* L. extract, variety Tygra, Extract D (CA-D)—*Cannabis sativa* L. extract, variety Dora, Rs—Spearman correlation coefficient (for non-parametric data distributions), *p*—statistical significance level, ASA—acetylsalicylic acid.

**Table 5:** Pearson correlations for the expression of genes (in the case of the right paw).

Group	Variable 1	Variable 2	Rp	*p*-Value
Control (rapeseed oil)	CB1 expression (right paw)	CB2 expression (right paw)	0.714	0.020
ASA	CB2 expression (right paw)	COX-1 expression (right paw)	0.694	0.038
CBD	CB1 expression (right paw)	COX-1 expression (right paw)	0.748	0.033

Rp—Pearson correlation coefficient (for parametric data distributions), *p*—statistical significance level, ASA—acetylsalicylic acid, CBD—cannabidiol.

**Table 6:** Positive Pearson correlations for the expression of genes vs. volumes/changes in volume.

Group	Variable 1	Variable 2	Rp	*p*-Value
Control (rapeseed oil)	Paw volume at 3 h (right paw)	TNFα expression (right paw)	0.889	0.001
Control (rapeseed oil)	Δ Paw volume (right–left) at 6 h	TNFα expression (right paw)	0.638	0.047
ASA	Δ Paw volume (right–left) at 3 h	COX-1 expression (right paw)	0.713	0.031
ASA	Δ Paw volume (right–left) at 1 h	COX-2 expression (right paw)	0.730	0.026
ASA	Paw volume at 6 h (right paw)	COX-2 expression (right paw)	0.728	0.026
ASA	Paw volume at 10 h (right paw)	TNFα expression (right paw)	0.672	0.048

Rp—Pearson correlation coefficient (for parametric data distributions), *p*—statistical significance level, Δ paw volume—difference between right and left paw volumes, ASA—acetylsalicylic acid.

**Table 7:** Negative Pearson correlations for the expression of genes vs. volumes/changes in volume.

Group	Variable 1	Variable 2	Rp	*p*-Value
CBD	Paw volume at 0 h (right paw)	CB1 expression (right paw)	−0.792	0.011
CBD	Paw volume at 0 h (right paw)	COX1 expression (right paw)	−0.770	0.025
Extract B (Tygra)	Paw volume at 1 h (right paw)	COX1 expression (right paw)	−0.900	0.039
Extract B (Tygra)	Δ Paw volume (right–left) at 3 h	COX1 expression (right paw)	−0.966	0.007

CBD—cannabidiol, Extract B (CA-B)—*Cannabis sativa* L. extract, variety Tygra, Rp—Pearson correlation coefficient (for parametric data distributions), *p*—statistical significance level, Δ paw volume—difference between right and left paw volumes.

## Data Availability

The data presented in this study are available on request from the corresponding author.

## Abbreviations

The following abbreviations are used in this manuscript:

ANOVA	Analysis of Variance
ASA	Acetylsalicylic Acid
CB1	Cannabinoid Receptor Type 1
CB2	Cannabinoid Receptor Type 2
CBD	Cannabidiol
CBD-A	Cannabidiolic acid
CGRP	Calcitonin Gene-Related Peptide
COX-1	Cyclooxygenase-1
COX-2	Cyclooxygenase-2
ECS	Endocannabinoid System
GAPDH	Glyceraldehyde-3-Phosphate Dehydrogenase
HSD	Honestly Significant Difference
IL1β	Interleukin 1 Beta
IL6	Interleukin 6
i.g.	Intragastric Administration
mRNA	Messenger Ribonucleic Acid
NFκB	Nuclear Factor Kappa B
NSAIDs	Non-Steroidal Anti-Inflammatory Drugs
PPARγ	Peroxisome Proliferator-Activated Receptor Gamma
qPCR (qRT-PCR)	Quantitative Real-Time Polymerase Chain Reaction
Rs	Spearman’s Rank Correlation Coefficient
Rp	Pearson’s Correlation Coefficient
∆9-THC-A	∆9-Tetrahydrocannabinolic acid
TNFα	Tumor Necrosis Factor α
TRPV1	Transient Receptor Potential Vanilloid Type 1
